# Transparency-enhancing technology allows the three-dimensional assessment of esophageal carcinoma obtained by endoscopic submucosal dissection

**DOI:** 10.1007/s10388-024-01055-x

**Published:** 2024-03-18

**Authors:** Yuichi Asahina, Munetoshi Hinata, Asami Tanaka, Kaori Oshio, Haruki Ogawa, Makoto Aihara, Hiroshi Onodera, Tetsuo Ushiku

**Affiliations:** 1 Department of Ophthalmology, Graduate School of Medicine, The University of Tokyo, 7‐3‐1, Hongo, Bunkyo‐ku, Tokyo, 113‐8655 Japan; 2 Department of Pathology, Graduate School of Medicine, The University of Tokyo, 7‐3‐1, Hongo, Bunkyo‐ku, Tokyo, 113‐8655 Japan; 3 Photon Science Center, The University of Tokyo, 7‐3‐1, Hongo, Bunkyo‐ku, Tokyo, 113‐8656 Japan; 4 Institute for Photon Science and Technology, The University of Tokyo, 7‐3‐1, Hongo, Bunkyo‐ku, Tokyo, 113‐8656 Japan; 5 Department of Gastroenterology, The University of Tokyo, 7‐3‐1, Hongo, Bunkyo‐ku, Tokyo, 113‐8655 Japan; 6 Department of Surgery, The University of Tokyo, 7‐3‐1, Hongo, Bunkyo‐ku, Tokyo, 113‐8655 Japan

**Keywords:** Esophageal carcinoma, Transparency-enhancing technology, Virtual hematoxylin and eosin, Microvessel morphology, 3D imaging

## Abstract

**Background:**

Although much progress has been made in diagnosis of carcinomas, no established methods have been confirmed to elucidate their morphological features.

**Methods:**

Three-dimensional structure of esophageal carcinomas was assessed using transparency-enhancing technology. Endoscopically resected esophageal squamous cell carcinoma was fluorescently stained, optically cleared using a transparency-enhancing reagent called LUCID, and visualized using laser scanning microscopy. The resulting microscope images were converted to virtual HE images for observation using ImageJ software.

**Results:**

Microscopic observation and image editing enabled three-dimensional image reconstruction and conversion to virtual HE images. The structure of abnormal blood vessels in esophageal carcinoma recognized by endoscopy could be observed in the 3 dimensions. Squamous cell carcinoma and normal squamous epithelium could be distinguished in the virtual HE images.

**Conclusions:**

The results suggested that transparency-enhancing technology and virtual HE images may be feasible for clinical application and represent a novel histopathological method for evaluating endoscopically resected specimens.

**Supplementary Information:**

The online version contains supplementary material available at 10.1007/s10388-024-01055-x.

## Introduction

The Japan Esophageal Society proposed a magnifying endoscopic diagnosis of superficial esophageal carcinoma based on microvessel morphology [1]. Although 3D endoscopic ultrasound enables 3D visualization of tissues, the image resolution is not sufficiently high for histopathological assessment. The ilLUmination of Cleared organs to IDentify target molecules (LUCID) method is a transparency-enhancing method using 2,2-thiodiethanol-based reagent [2–4]. Here, we aimed to observe 3D structure of endoscopically resected esophageal carcinomas using LUCID.

## Materials and methods

Endoscopically resected esophageal carcinoma specimens from three patients were selected. All specimens included squamous cell carcinomas invading the submucosal layer (pT1b). The resected specimens were fixed in formalin (20% neutral buffered formalin), stained with iodine for gross examination, cut with 3–4 mm width and stored formalin-fixed, paraffin-embedded (FFPE). These FFPE specimens were deparaffinized and immersed in 1 × Tris-buffer saline (TBS) (Nippon Gene Co., Toyama, Japan) with 1 µg/mL 4′,6-diamidino-2-phenylindole (DAPI) (Dojindo Molecular Technologies, Kumamoto, Japan) and 5 µg/mL *Lycopersicon esculentum* lectin conjugated with DyLight 594 (Vector Laboratories, Burlingame, CA) for 48 h. The samples were then washed with 1 × TBS and immersed in LUCID (PhotonTech Innovatsions Co., Ltd., Tokyo, Japan) for over 48 h.

The samples were imaged using a confocal microscope (FV10i-LIV; Olympus, Tokyo, Japan) and multiphoton-excited fluorescence microscope (A1MP^+^; Nikon). Images of the horizontal sections from each sample were stacked and saved as single-tag image files. In this study, it took 10 s to capture a 2D image of a 500 µm square area with 10 × magnifying lens of a confocal microscope. Therefore, for example, it took about 30 min to obtain a 3D image of a 1000 µm square taken every 10 µm up to 500 µm. The image files were analyzed using ImageJ (http://rsb.info.nih.gov/ij/) and NIS-Elements Advanced Research software (Nikon). Images of the nuclei, extracellular matrix and cytoplasm were respectively observed at 430–460 nm, 520–560 nm, and 610–630 nm. Virtual HE-stained images were created using ImageJ based on the intensity values of the scanned HE stained images.

## Results

**Fig. 1:**
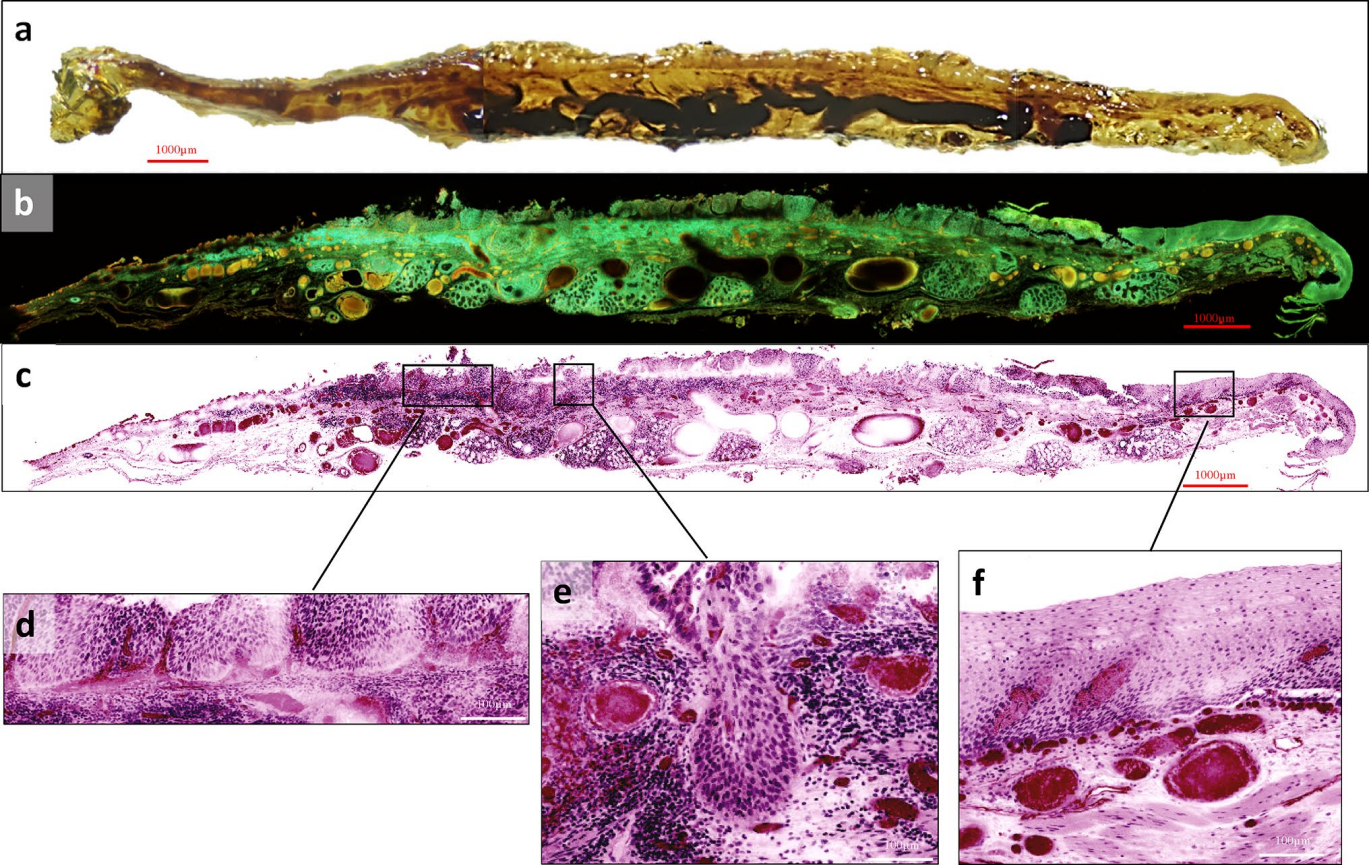
Macroscopic and microscopic views of the optically cleared esophagus and its virtual HE image obtained from Case #1. **a** Macroscopic view of the optically cleared esophagus specimen obtained by ESD. This figure is a cut surface of the specimen; **b** fluorescent image of (**a**) obtained with a two-photon microscope; **c–f** virtual HE images converted from (**b)**. **d–f** Magnified virtual HE images. **d** and **e** Are taken from the same specimen as (**c**), but at a slightly deeper depth. Squamous cell carcinoma can be seen in (**d**) and (**e**), and the tumor invading into the depth can also be seen in (**e**). Normal epithelium can be seen in (**f**). *HE* hematoxylin and eosin, *ESD* endoscopic submucosal dissection

The transparency of the tissue was sufficiently enhanced for the blood vessels in the specimen to be detectable upon gross examination. Figure 1 shows a macroscopic image of an optically cleared esophagus, a fluorescent image, and virtual HE images using the procedure mentioned above. Squamous cell carcinoma (Fig. 1d, e) and normal squamous epithelium (Fig. 1f) can be distinguished in the virtual HE images.

**Fig. 2:**
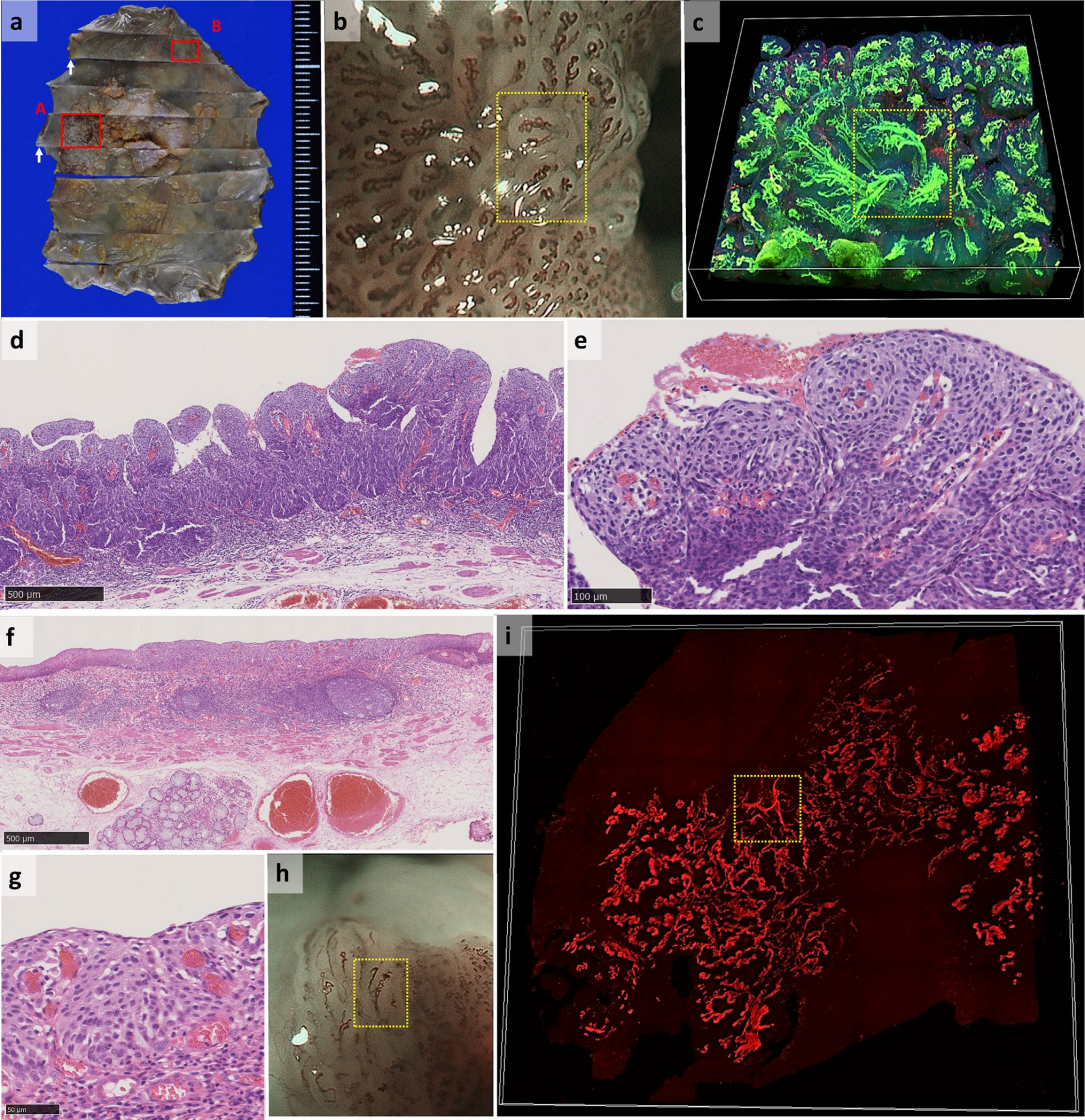
Macroscopic view of the ESD specimen, endoscopic view of its blood vessels, and 3D constructed image of blood vessels and original HE and virtual HE images obtained from Case #2. **a** Macroscopic view of the esophageal specimen obtained by ESD. White arrows indicate the direction where the tissue section was sliced using a microtome; **b** magnified endoscopic NBI of the red square area (A) in (**a**) showing Type B1 vessels; **c** 3D image of Type B1 vessels constructed from confocal microscopy images, with the yellow dotted squares of (**b**) and (**c**) indicating the same vessels. The scale is 5 mm square and 0.5 mm depth; **d** original HE staining image of the red square in (**a**); **e** A higher power magnified image of (**d**); **f** original HE-stained image of the red square (B) in (**a**); **g** a higher power magnified image of (**f**); **h** magnified endoscopic NBI of the red square (B) in (**a**) showing Type B2 vessels; **i** 3D image of Type B2 vessels constructed from confocal microscopy images, with the yellow dotted squares in (**h**) and (**i**) indicating the same vessels. *ESD*, endoscopic submucosal dissection. *HE* hematoxylin and eosin, *NBI* narrow band image

Figures 2 shows macroscopic and microscopic views of another specimen. This specimen had Type B1 and B2 vessels that were endoscopically detected. B1 vessels are defined as abnormal microvessels with a loop-like formation, whereas B2 vessels are without a loop-like formation that have elongated transformation [1]. A papillary structure with B1 vessels is shown in Fig. 2c, whereas in Fig. 2i, B2 vessels showing a stretched transformation without a loop-like formation can be seen in contrast to the normal intrapapillary capillary loops on the right side.

**Fig. 3:**
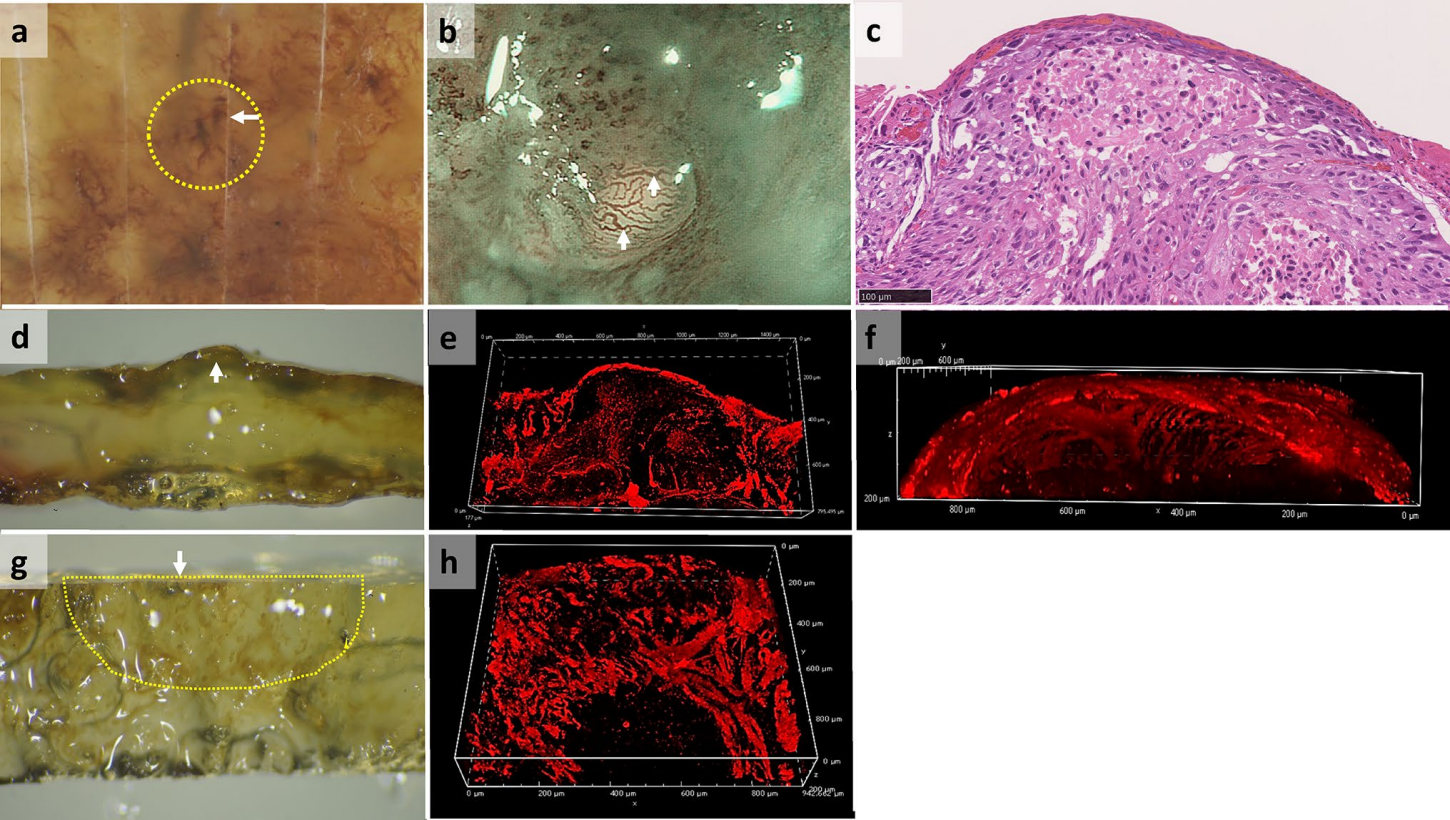
Macroscopic view of the original ESD and optically cleared specimen, endoscopic view and 3D constructed images of its blood vessels, and original HE stained section obtained from Case #3. **a** Macroscopic view of the esophageal specimen obtained by ESD, with an white arrow indicating the direction where the tissue section was sliced using a microtome; **b** magnified endoscopic NBI of the yellow dotted area in (**a**) showing Type B2-like vessels (indicated with an white arrow); **c** original HE-stained image of the yellow dotted area in (**a**); **d** macroscopic view of the yellow dotted area in (**a**) after optical clearance, as observed from the same direction as in (**c**), (**e**) and (**f**), with an white arrow indicating blood vessels which look like “a blood pool”; **e–f** 3D images of blood vessels constructed from confocal microscopy images in the same direction as in (**c**) and (**d**); **f** top dome-shaped area of (**e**); **g** macroscopic view of the yellow dotted area in (**a**) after optical clearance, as observed from the same direction as in (**a**) and (**b**), with an white arrow indicating the same cut surface as the one in (**a**). A dotted area showing a bulge observed in (**b**); **h** 3D image of blood vessels constructed from confocal microscopy images in the same direction as (**g**). *ESD* endoscopic submucosal dissection, *HE* hematoxylin and eosin, *NBI* narrow band image

Figure 3 shows the macroscopic and microscopic views of the other specimen. This case was determined to have Type B2 vessels on endoscopy prior to ESD, and the superficial vessels on the HE-stained image alone appeared to be blood pools rather than blood vessels. However, upon transparency, the vascular structure was visible on gross examination, and the 3D reconstructed images revealed that these vessels were not strictly B2 vessels elongating from inside the tumor but rather superficial vessels pushed up into a dome shape by the tumor, mimicking B2 vessels.

## Discussion

In this study, we aimed to assess the 3D structure of endoscopically resected esophageal carcinoma using a transparency-enhancing reagent that enables an arbitrary cross-section of the specimen to be observed and reconstructed in 3 dimensions, especially the 3D structure of B1 and B2 vessels. Interestingly, we found a case in which irregular vessels diagnosed as B2 vessels on endoscopy were revealed to be superficial vessels mimicking B2 vessels. The limitations of this study are the lack of quantitative evaluation, the inability to evaluate vascular invasion with virtual HE staining, and the small number of cases evaluated. The transparency-enhancing method using pathology specimens is, to the best of our knowledge, still unprecedented, so we do not believe it is immediately applicable to clinical use and is a subject for future research. However, this method could enable to more accurately assess tumor depth, vascular invasion, and margin status and detect abnormal vascular structures by evaluating at any virtual slice. Future work is warranted on this technology.

### Supplementary Information

Below is the link to the electronic supplementary material.

Supplementary file1 Supplementary file1 Supplemental Table 1. Histological factors according to the classification of Japan Esophageal Society. Supplemental Figure 1. Original HE stained sections of the deepest part of carcinomas before optical clearing obtained from three patients. (a) HE staining of the esophageal carcinoma from Case #1; (b) Higher-power magnified image of (a). Vertical cut margin is positive for carcinoma; (c) HE staining of the esophageal carcinoma from Case #2; (d) Higher-power magnified image of (c). Vertical cut margin is negative for carcinoma; (e) HE staining of the esophageal carcinoma from Case #3; (f) Higher-power magnified image of (e). Vertical cut margin is negative for carcinoma. The scales are 500µm in (a), (c), (e) and 100µm in (b), (d), (f). HE: hematoxylin and eosin. Supplemental Figure 2. A schema of procedure. FFPE: formalin-fixed paraffin-embedded. ESD: endoscopic submucosal dissection. HE: hematoxylin and eosin. LUCID: ilLUmination of Cleared organs to IDentify target molecules. Supplemental Figure 3. Macroscopic view of the ESD specimen and its 3D constructed image of blood vessels and original HE and virtual HE images obtained from Case #2. (a) Original HE-stained image of the deepest part of carcinoma; (b) A higher power magnified image of the yellow square (A) in Fig. a; (c) 3D image of the specimen constructed from confocal microscopy images, showing the same area as Fig. a: (d) 3D image of the blood vessels constructed from confocal microscopy images, showing the same area as the yellow square (B) in Fig. a. ESD: endoscopic submucosal dissection. HE: hematoxylin and eosin. Supplemental Figure 4. Microscopic images of sections from the specimen stained with HE and anti-D2-40 before and after optical clearing obtained from Case #1. (a) HE staining before optical clearing; (b) D2-40 immunostaining before optical clearing; (c) HE staining after optical clearing; (d) D2-40 immunostaining after optical clearing. The morphology of the samples was well-preserved, and the quality of the staining was sufficient for conventional histopathological examination, which was assessed by two pathologists. HE: hematoxylin and eosin. Supplemental video clip 1. 3D reconstruction of blood vessels of esophageal carcinoma seen from the cut surface shown in Fig. 3e. To facilitate identification of the location of blood vessels in the tissue, the movie includes not only blood vessels but also other tissues. Nuclei are depicted in blue (DAPI), and blood vessels and blood are depicted in red (lectin) and green (autofluorescence). Stretched blood vessels seen at the apex of the tumor are continuous from subepithelial capillary network (SECN) in the stroma between intraepithelial carcinoma nests and subepithelial invasive cancer nests. Supplemental video clip 2. 3D reconstruction of blood vessels of esophageal carcinoma seen from the same direction as endoscopy shown in Fig. 3h. Blood vessels are pushed up by the tumor and forming a dome shape (PPTX 47089 KB)
