# Supplementary material for: Transparency-enhancing technology allows the three-dimensional assessment of esophageal carcinoma obtained by endoscopic submucosal dissection
Source: Esophagus. 2024 Mar 18;21(3):405–9. doi: 10.1007/s10388-024-01055-x (PMC11199231; doi:10.1007/s10388-024-01055-x)
Supplement: Supplementary file 1 — Supplementary file1 Supplementary file1 Supplemental Table 1. Histological factors according to the classification of Japan Esophageal Society. Supplemental Figure 1. Original HE stained sections of the deepest part of carcinomas before optical clearing obtained from three patients. (a) HE staining of the esophageal carcinoma from Case #1; (b) Higher-power magnified image of (a). Vertical cut margin is positive for carcinoma; (c) HE staining of the esophageal carcinoma from Case #2; (d) Higher-power magnified image of (c). Vertical cut margin is negative for carcinoma; (e) HE staining of the esophageal carcinoma from Case #3; (f) Higher-power magnified image of (e). Vertical cut margin is negative for carcinoma. The scales are 500µm in (a), (c), (e) and 100µm in (b), (d), (f). HE: hematoxylin and eosin. Supplemental Figure 2. A schema of procedure. FFPE: formalin-fixed paraffin-embedded. ESD: endoscopic submucosal dissection. HE: hematoxylin and eosin. LUCID: ilLUmination of Cleared organs to IDentify target molecules. Supplemental Figure 3. Macroscopic view of the ESD specimen and its 3D constructed image of blood vessels and original HE and virtual HE images obtained from Case #2. (a) Original HE-stained image of the deepest part of carcinoma; (b) A higher power magnified image of the yellow square (A) in Fig. a; (c) 3D image of the specimen constructed from confocal microscopy images, showing the same area as Fig. a: (d) 3D image of the blood vessels constructed from confocal microscopy images, showing the same area as the yellow square (B) in Fig. a. ESD: endoscopic submucosal dissection. HE: hematoxylin and eosin. Supplemental Figure 4. Microscopic images of sections from the specimen stained with HE and anti-D2-40 before and after optical clearing obtained from Case #1. (a) HE staining before optical clearing; (b) D2-40 immunostaining before optical clearing; (c) HE staining after optical clearing; (d) D2-40 immunostaining after optical clearing. The morpholog [file 10388_2024_1055_MOESM1_ESM.pptx]

## Slide 1
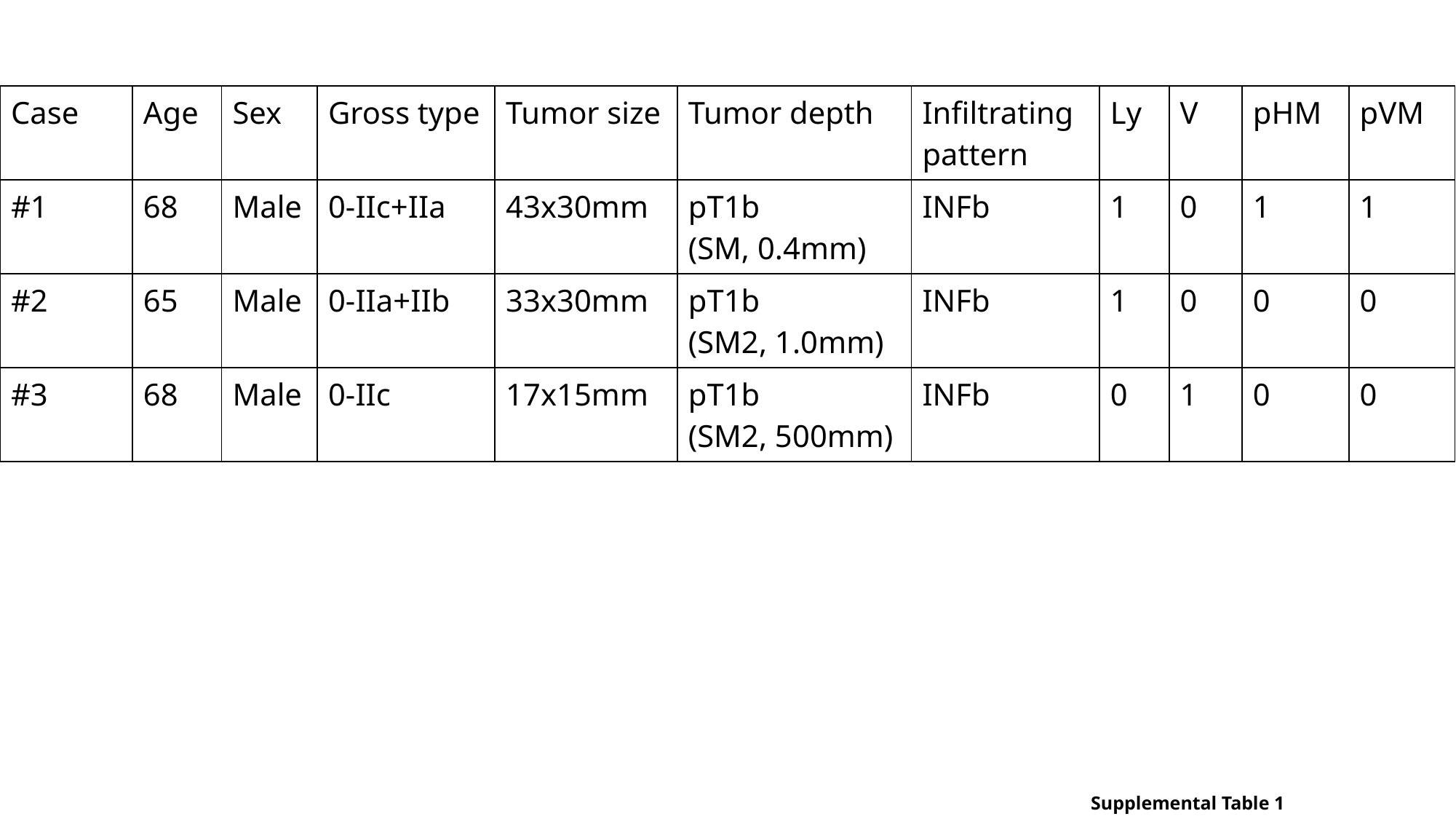

| Case | Age | Sex | Gross type | Tumor size | Tumor depth | Infiltrating pattern | Ly | V | pHM | pVM |
| --- | --- | --- | --- | --- | --- | --- | --- | --- | --- | --- |
| #1 | 68 | Male | 0-IIc+IIa | 43x30mm | pT1b (SM, 0.4mm) | INFb | 1 | 0 | 1 | 1 |
| #2 | 65 | Male | 0-IIa+IIb | 33x30mm | pT1b (SM2, 1.0mm) | INFb | 1 | 0 | 0 | 0 |
| #3 | 68 | Male | 0-IIc | 17x15mm | pT1b (SM2, 500mm) | INFb | 0 | 1 | 0 | 0 |
Supplemental Table 1

## Slide 2
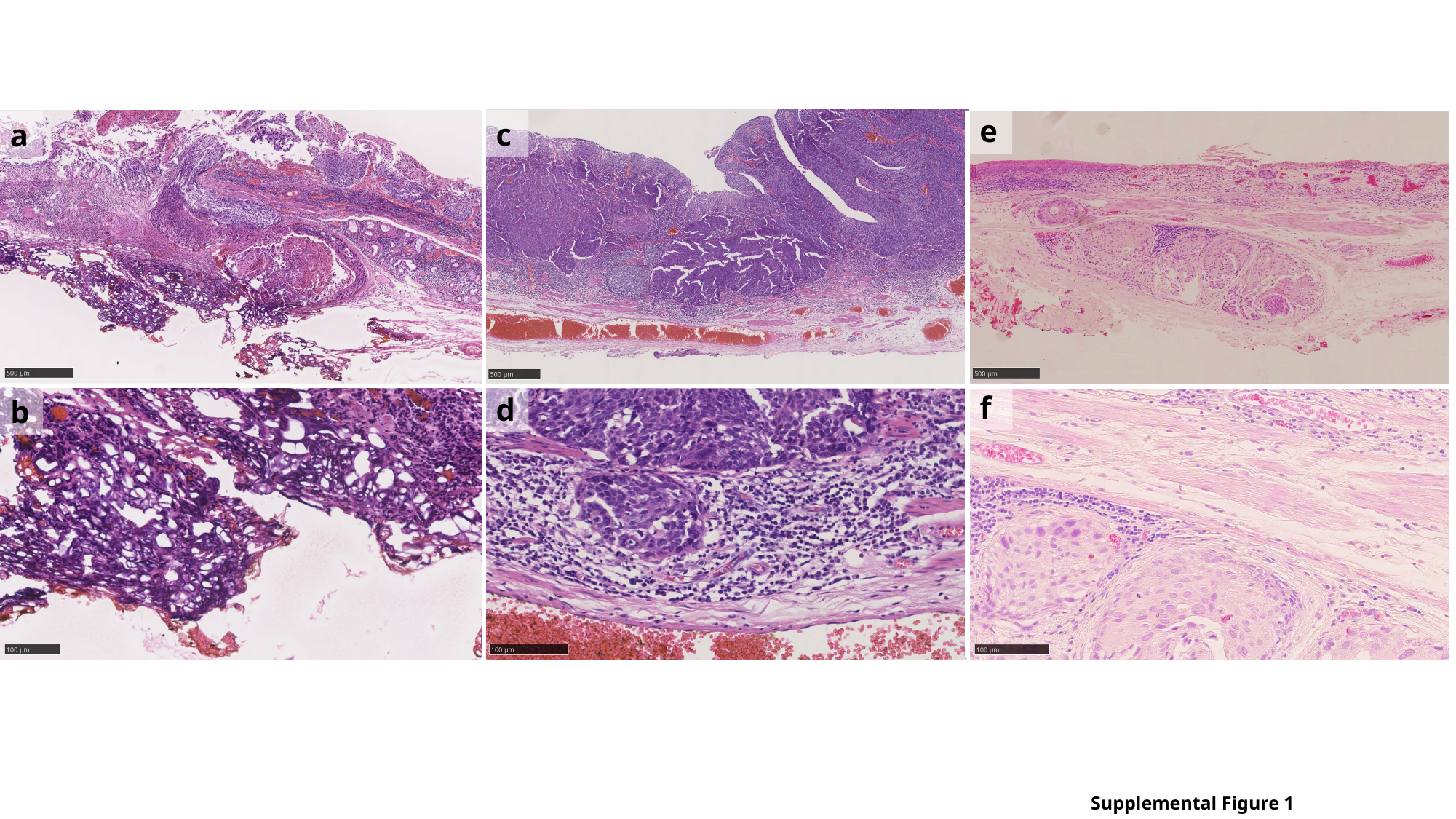

e
c
a
f
d
b
Supplemental Figure 1

## Slide 3
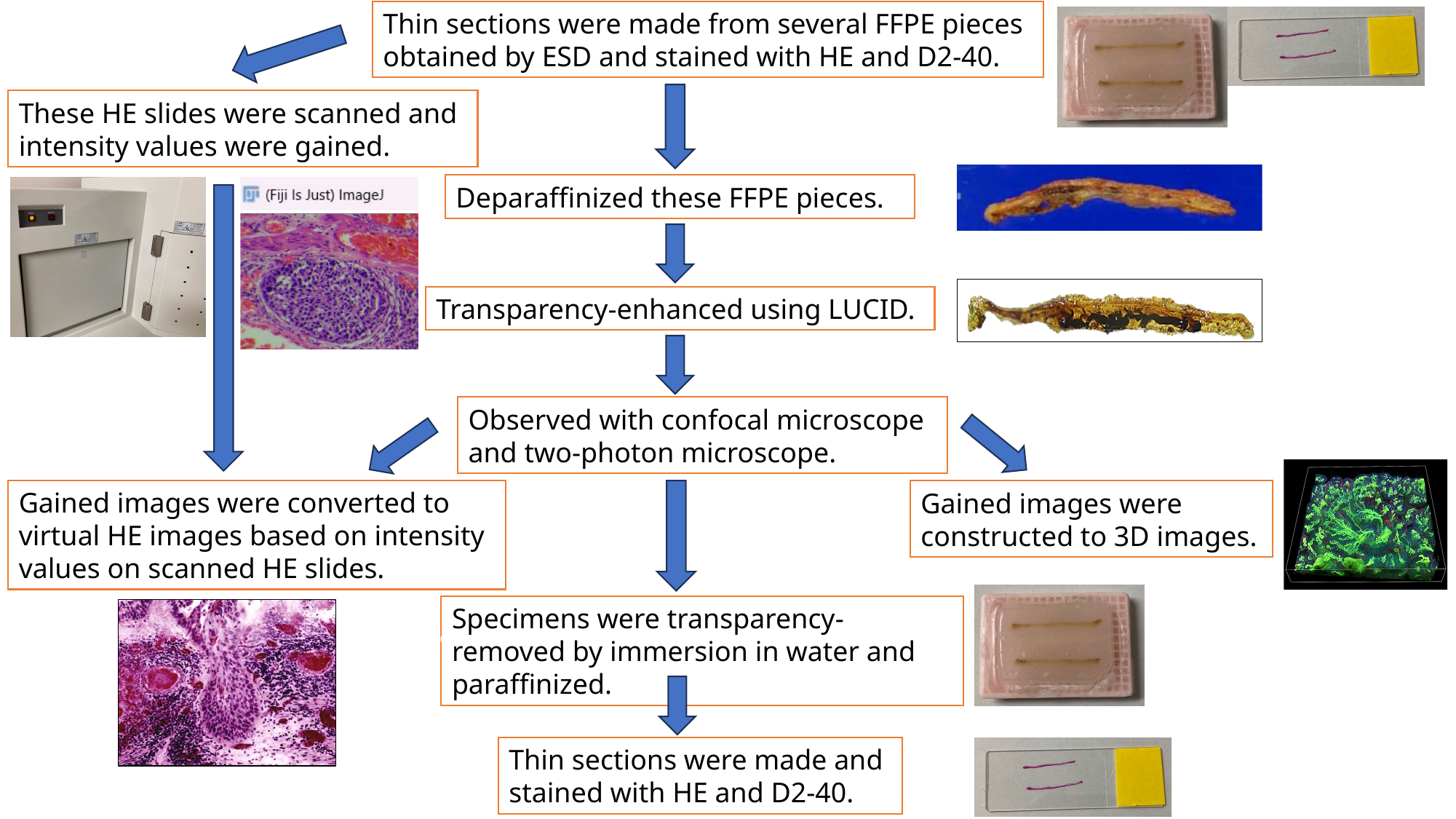

Supplemental Figure 2
Thin sections were made from several FFPE pieces obtained by ESD and stained with HE and D2-40.
These HE slides were scanned and intensity values were gained.
Deparaffinized these FFPE pieces.
Transparency-enhanced using LUCID.
Observed with confocal microscope and two-photon microscope.
Gained images were converted to virtual HE images based on intensity values on scanned HE slides.
Gained images were constructed to 3D images.
Specimens were transparency-removed by immersion in water and paraffinized.
Thin sections were made and stained with HE and D2-40.

## Slide 4
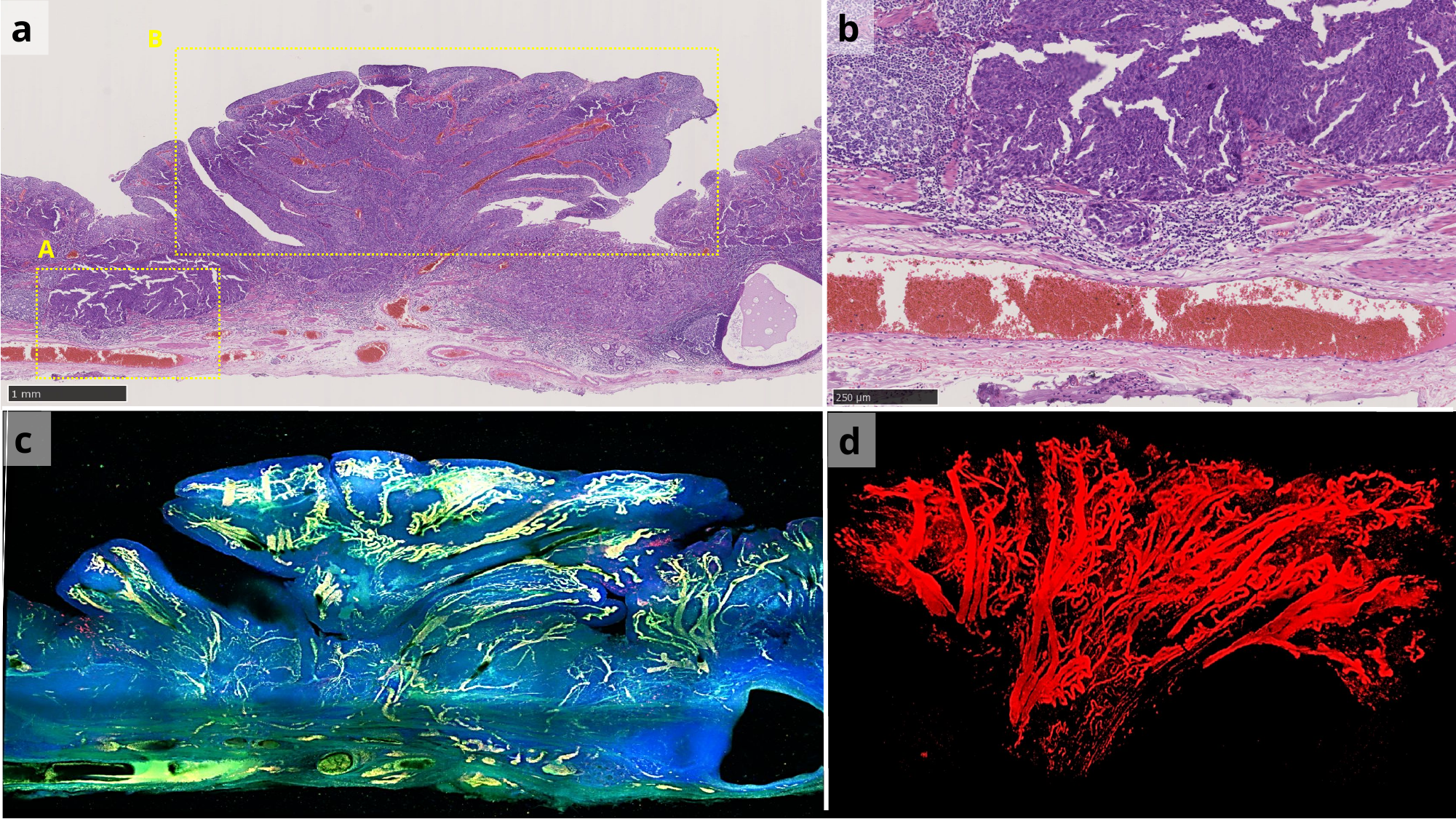

Supplemental Figure 3
b
a
B
A
c
d

## Slide 5
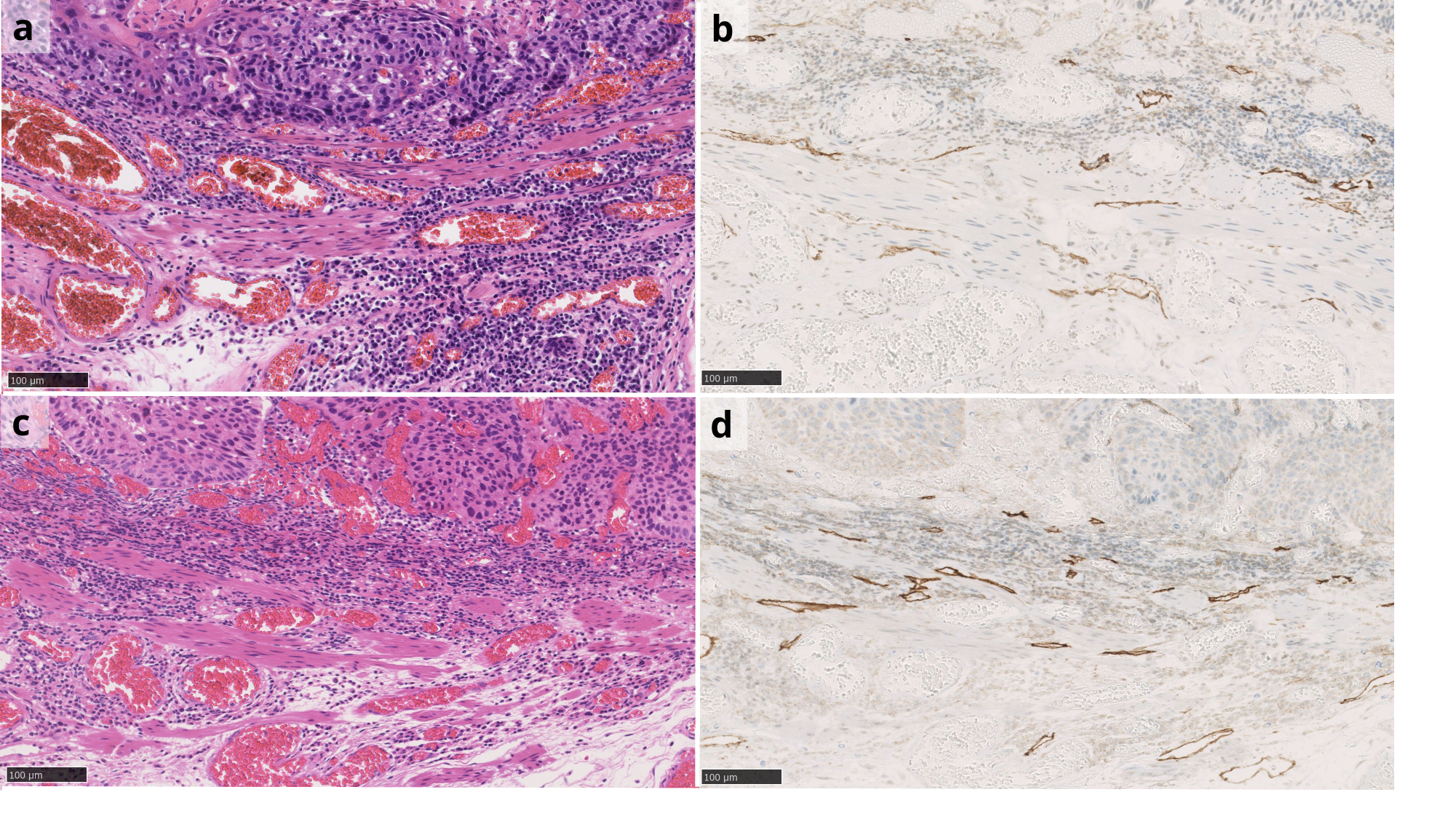

Supplemental Figure 4
a
b
c
d

## Slide 6
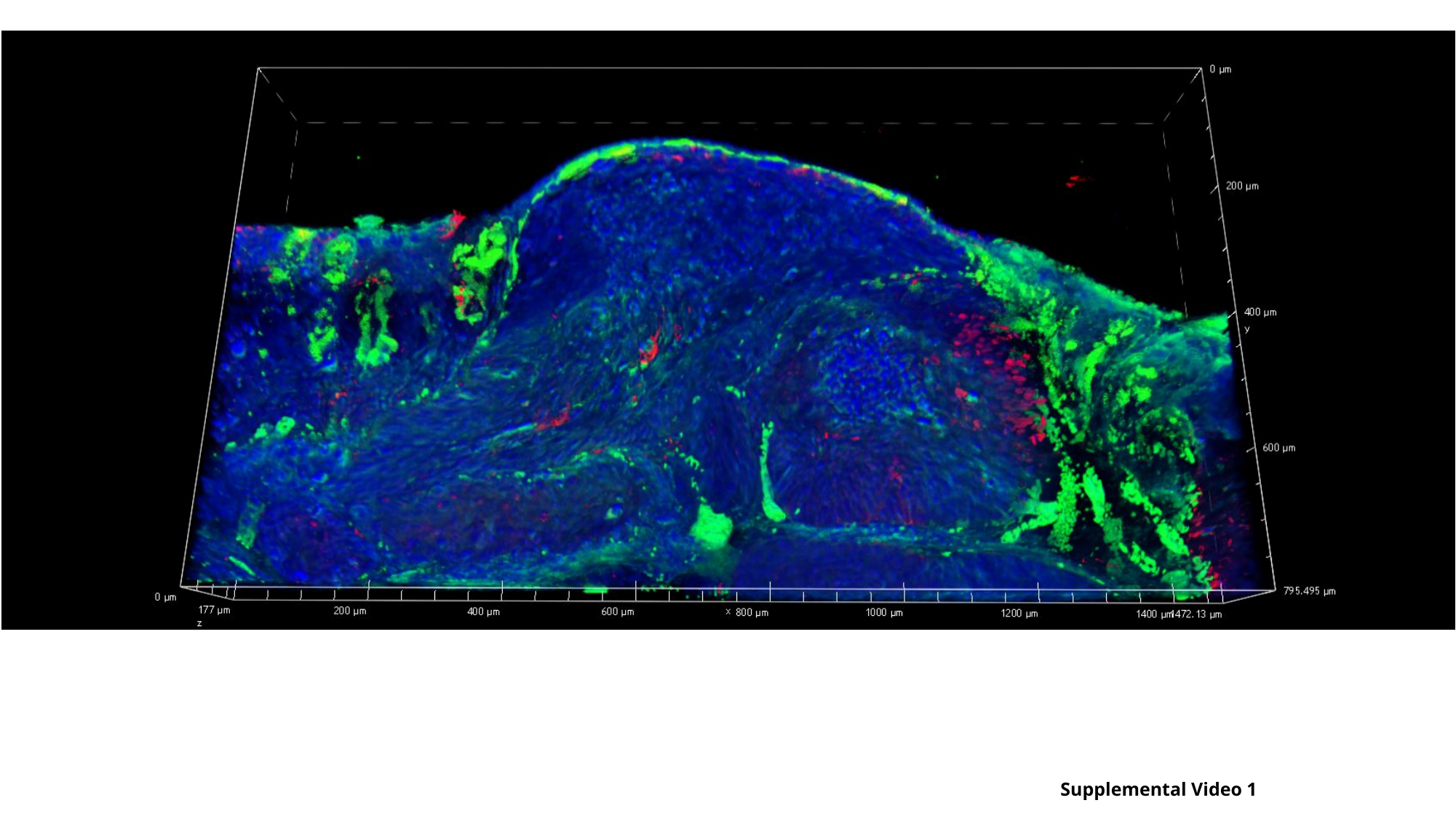

Supplemental Video 1

## Slide 7
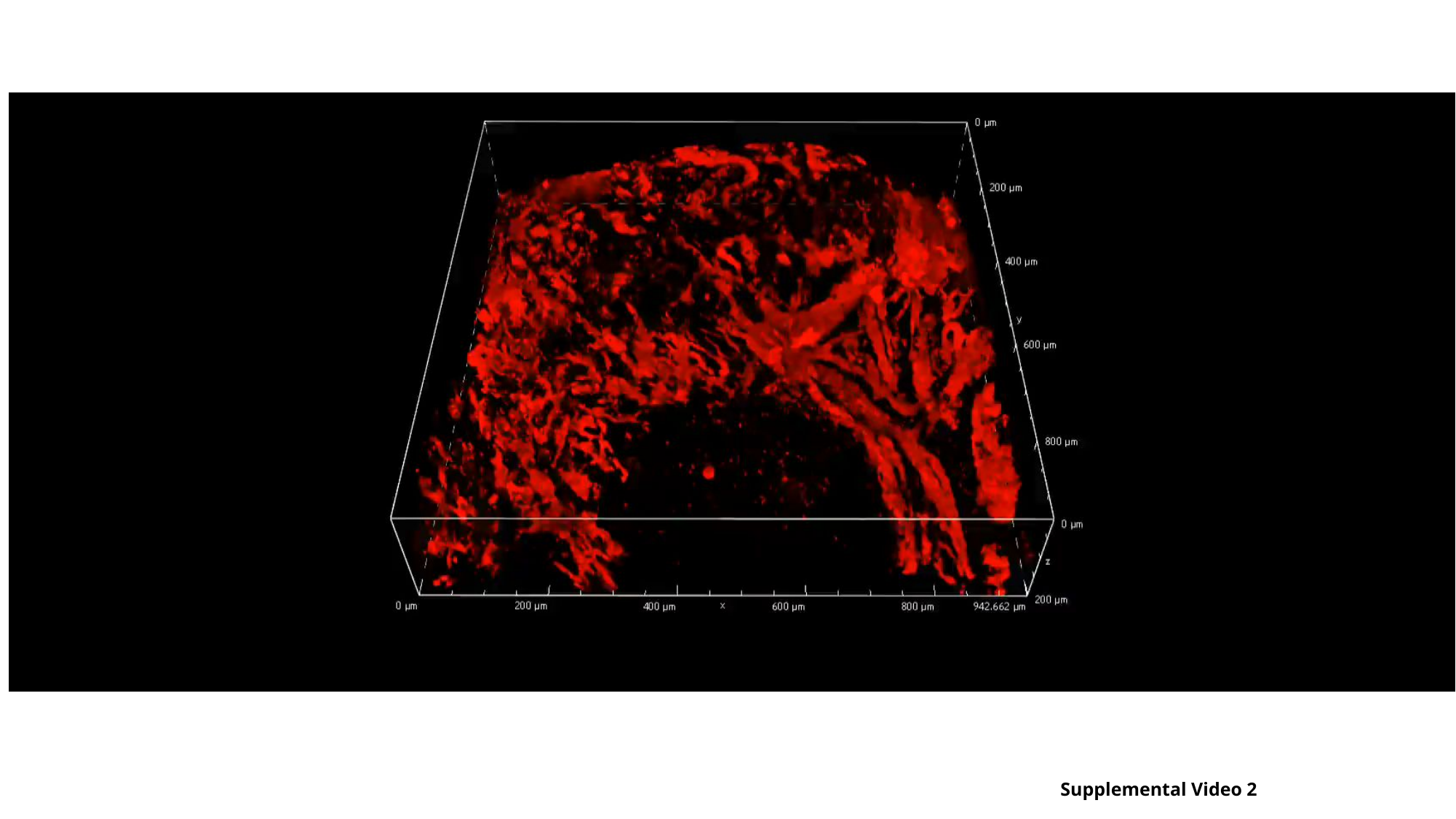

Supplemental Video 2
